# Crosstalk between endoplasmic reticulum stress and multidrug-resistant cancers: hope or frustration

**DOI:** 10.3389/fphar.2023.1273987

**Published:** 2023-09-18

**Authors:** Bowen Qing, Song Wang, Yingan Du, Can Liu, Wei Li

**Affiliations:** ^1^ First Affiliated Hospital of Hunan Normal University, Department of Hematology, Hunan Provincial People’s Hospital, Changsha, China; ^2^ Department of Neurology, Xiangya Hospital, Central South University, Changsha, China

**Keywords:** endoplasmic reticulum stress, unfolded protein response, multidrug resistance, apoptosis, molecular mechanism

## Abstract

Endoplasmic reticulum stress (ERS) is a kind of cell response for coping with hypoxia and other stresses. Pieces of evidence show that continuous stress can promote the occurrence, development, and drug resistance of tumors through the unfolded protein response. Therefore, the abnormal ac-tivation of ERS and its downstream signaling pathways not only can regulate tumor growth and metastasis but also profoundly affect the efficacy of antitumor therapy. Therefore, revealing the molecular mechanism of ERS may be expected to solve the problem of tumor multidrug resistance (MDR) and become a novel strategy for the treatment of refractory and recurrent tumors. This re-view summarized the mechanism of ERS and tumor MDR, reviewed the relationship between ERS and tumor MDR, introduced the research status of tumor tissue and ERS, and previewed the prospect of targeting ERS to improve the therapeutic effect of tumor MDR. This article aims to provide researchers and clinicians with new ideas and inspiration for basic antitumor treatment.

## 1 Introduction

Malignant tumors are one of the most important public health problems in the world (Lin et al., 2021b). In the United States, 1,958,310 new cases and 609,820 die cases of invasive tumors are estimated to occur in 2023. The overall cancer incidence and mortality rates were 488.2/100,000 and 177.5/100,000 in males and 423.3/100,000 and 128.7/100,000 in females during 2020 (Siegel et al., 2023). China is the largest developing country in the world. According to statistics, China recorded an estimated 4,820,000 new cancer cases in 2022. China is the largest developing country in the world. According to statistics, China recorded an estimated 4,820,000 new cancer cases in 2022. In 2020, the overall cancer incidence in China was estimated to be 204.8/100,000. The cancer mortality rate was 163.9/100,000 in males and 98.1/100,000 in females (Qiu et al., 2021; Xia et al., 2022). Malignant tumors are not only a medical but also a complex problem composed of societal, family, and other aspects. With China’s economic development and the increase in life expectancy, the rise in cancer patients is not an unexplained problem. Notably, the number of cancer deaths in China was five times higher than that in the United States in the same year. This phenomenon may not only be due to the level of medical care and decision-making but also the result of the economic situation of patients and the availability of medical resources. The treatment of cancer has consistently been a major concern worldwide. In recent decades, with the rapid development in genetics and molecular biology, cancer treatment has gradually shifted toward precision medicine at the individual patient level. A personalized approach based on individual cancer genomic information has the potential to identify clinically viable target molecules and aid in the selection of appropriate therapies for individual patients. However, given the limited number of molecular targeted drugs, only a small proportion of patients can benefit from genetic analysis. Drug therapy remains the most important component of cancer treatment, especially for patients who have lost the opportunity to undergo surgery at the time of diagnosis. Drug therapy agents include chemotherapeutic agents, targeted agents, and immune checkpoint inhibitors. The continuous development of drug therapy has resulted in inspiration and motivation for the cure of tumors. However, the multidrug resistance (MDR) of tumor cells led to serious challenges in human survival. MDR refers to the cross-resistance of cancer cells to anticancer drugs with different structures and mechanisms of action (Assaraf et al., 2019). MDR is responsible for more than 90% of deaths in cancer patients receiving conventional chemotherapy or new targeted agents (Bukowski et al., 2020). Despite the billions of dollars that have been invested in tumor resistance research and the development of new anticancer therapies, MDR remains the greatest obstacle to tumor cure for patients without surgical opportunity. Chemotherapy, immunotherapy, or molecular targeted therapy may be effective initially, but surviving drug-resistant tumor cells will ultimately lead to tumor recurrence and treatment failure (Dhanyamraju et al., 2022).

The endoplasmic reticulum (ER) is an organelle in eukaryotic cells that plays a crucial role in protein folding, lipid biosynthesis, and calcium signaling. When cells experience stress conditions, such as nutrient deprivation, oxidative stress, and inflammatory responses, the capability of the ER to fold proteins is compromised. This results in the accumulation of unfolded or misfolded proteins in the ER cavity and triggers a signaling pathway called the unfolded protein response (UPR) (Ren et al., 2021). The UPR is a highly conserved cellular stress response pathway that alleviates endoplasmic reticulum stress (ERS). It involves the activation of three major signaling pathways, namely, protein kinase-like ER kinase (PERK), inositol-requiring enzyme 1α (IRE1α), and activating transcription factor 6 (ATF6) (Kaufman, 2002). The activation of these pathways is ultimately a transcriptional program that restores protein folding homeostasis, reduces protein synthesis, and increases protein degradation. However, if ERS persists and becomes extremely severe, the UPR signaling pathway will change its role from a cytoprotective one to a promoter of apoptosis (Chen and Cubillos-Ruiz, 2021). Despite the substantial evidence on the persistence of ERS and UPR activation in numerous forms of cancer, whether these processes ultimately inhibit or promote tumor growth in patients remains inconclusive (Oakes and Papa, 2015). On the one hand, a variety of drugs exert antitumor effects through ERS-related signaling pathways. On the other hand, ERS is also related to the mechanism of tumor MDR, and its regulation can affect tumor resistance to antitumor drugs or reverse drug resistance (Nie et al., 2021). Although considerable work is needed before this finding can be used clinically, it gives new hope to patients with refractory or recurrent tumors. Therefore, this article reviews the research progress on multidrug-resistant cancers and ERS to provide novel ideas for basic and clinical researchers.

## 2 Mechanism of ERS

**FIGURE 1:**
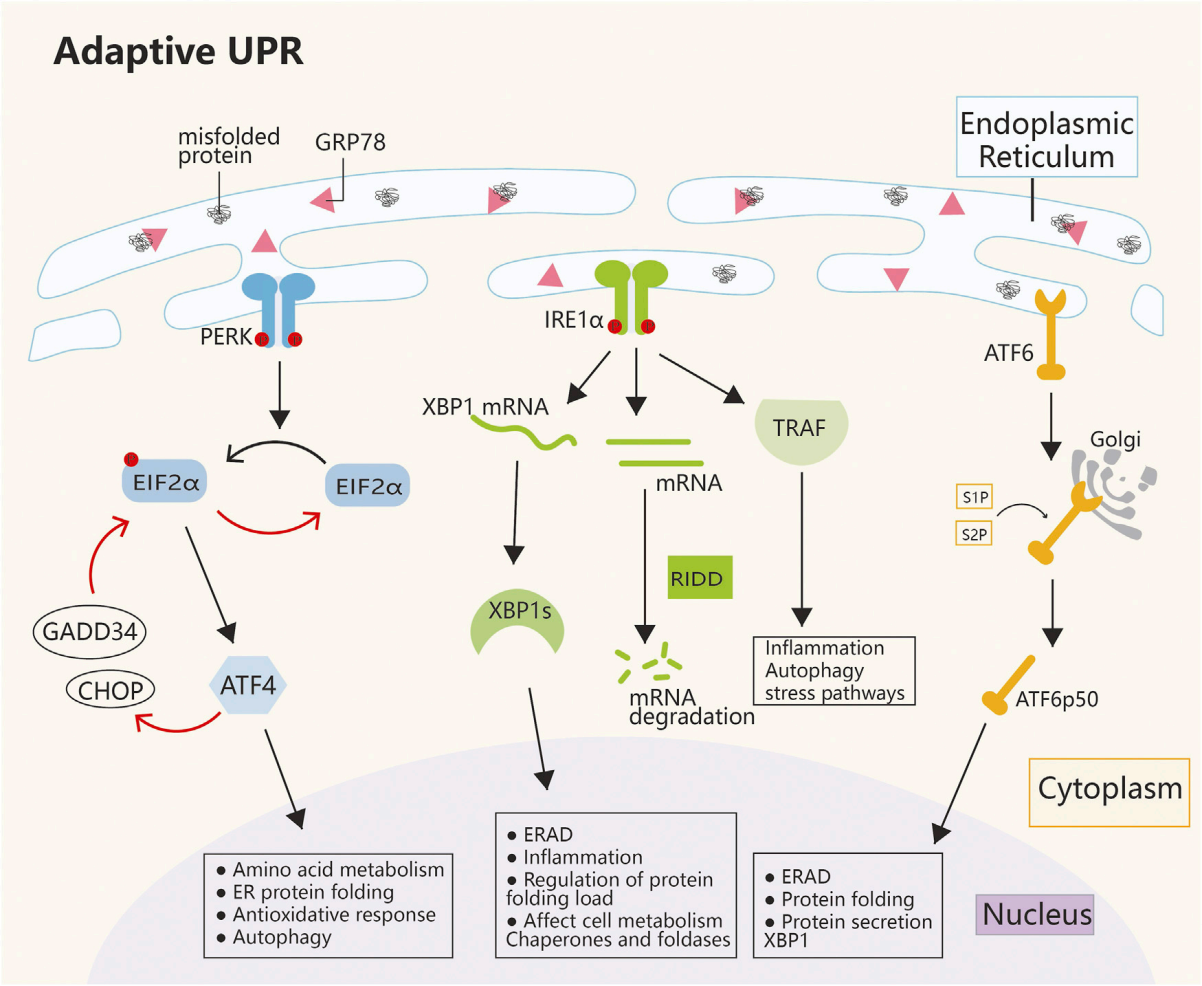
Mechanism of ERS. The main pathways of ERS are PERK, IRE1α, and ATF6. The three ERS sensors collaborate to coordinate the UPR signals. Under normal conditions, GRP78 is connected to the ERS sensor, which leaves it inactive. During ERS, GRP78 dissociates from the three transmembrane proteins on the ER membrane and activates their pathways. The IRE1α, PERK, and ATF6 pathways work together to regulate multiple genes, with the ultimate goal of restoring ER homeostasis and inducing cancer survival, angiogenesis, metastasis, and cell death resistance. ATF4, activating transcription factor 4; ATF6, activating transcription factor 6; CHOP, C/EBP homologous protein; EIF2α, eukaryotic initiation factor 2α; ER, endoplasmic reticulum; GADD34, growth arrest and DNA damage-inducible protein; GRP78, glucose-regulated protein 78; IRE1α, inositol-requiring enzyme 1α; PERK, protein kinase-like ER kinase; RIDD, regulated IRE1α-dependent decay; S1P, site-1 protease; S2P, site-2 protease; TRAF, tumor necrosis factor receptor-associated factor; XBP1, X-box binding protein 1.

Since the 1980s, cellular stress has been known to induce the expressions of molecular chaperones in the inner reticulum (Koch et al., 1986), but it was not until the 1990s, when the first ERS sensor (IRE1) was discovered, that researchers paid attention to the UPR. Subsequently, two ERS receptors have been identified, namely, ATF6 and PERK; in addition, three ER transmembrane proteins collectively regulate the functions of thousands of genes involved in ER control while also regulating the rate of protein synthesis (Marciniak et al., 2022). Over the past decade, cellular alterations secondary to ERS have increasingly been recognized as central factors in the pathophysiology of human diseases. ERS and persistent UPR signaling have been documented well in tissues affected by diabetes, neurodegeneration, stroke, pulmonary fibrosis, viral infections, inflammatory diseases, cancer, and heart diseases (Oakes and Papa, 2015). The ability to fold proteins within the ER varies by cell type and often depends on the size of the ER; still, regardless of the ER size, cells operate at load limits and often encounter situations that impose workload on the ER beyond its capacity (Tabas and Ron, 2011). Moreover, a wide range of cellular disorders, such as hypoxia, nutrient deficiency, and inflammation, can affect the efficiency of protein folding in the ER and lead to the accumulation of misfolded proteins in this organelle. When the ER protein folding capacity is overwhelmed, ERS is then triggered. The mechanism of ERS mainly depends on the duration and severity of stimulation, that is, restoration of homeostasis or apoptosis and necrosis (Oakes and Papa, 2015). Various physiological and pathological stimuli can cause ERS, which triggers the UPR that transmits information on protein folding status to the nucleus and cytoplasm to regulate the protein folding capacity of the cell (Hetz et al., 2020). When mild to moderate (but persistent) ERS occurs, cells induce transcriptional and translational changes through the homeostatic UPR (hUPR), which promotes cell adaptation and improves cell survival. However, when ERS progresses to the point where the hUPR is insufficient to restore homeostasis, the UPR in the cell becomes dominated by the terminal UPR. This process actively initiates apoptosis and prevents sustained cell damage (Nie et al., 2021). The UPR is initiated by three ER-resident transmembrane proteins: IRE1α, PERK, and ATF6. In the absence of stress, these transmembrane proteins are inactivated when they bind to the ER lumen molecular chaperone glucose-regulated protein 78 (GRP78). During stress condition, the amount of unfolded proteins in the ER lumen increases, which triggers the dissociation of the three protein receptors from GRP78, which in turn activates downstream signaling pathways in a cascade (Fu et al., 2022b). Once activated, the three parallel UPR signaling pathways alter the rate of protein synthesis and trafficking to the ER through autophagy and ER-associated protein degradation (ERAD) pathways, regulate protein folding and maturation and quality control, and participate in protein trafficking and elimination of misfolded proteins (Hetz et al., 2020) (Figure 1).

### 2.1 PERK pathway

PERK is a transmembrane protein kinase on the ER membrane with serine/threonine protein kinase activity at the cytoplasmic terminus. When activated by the UPR, PERK dimerizes itself and phosphorylates eukaryotic translation initiation factor 2α (eIF2α). After phosphorylation, eIF2α is inactivated, and subsequent mRNA translation is arrested, which reduces the ER load (Starck et al., 2016; Wang et al., 2022). Phosphorylated eIF2α can selectively activate ATF4. As a transcription factor (TF), ATF4 can regulate a wide range of target genes; thereby, it indirectly regulates cellular antioxidative stress, the rate of protein synthesis, and cell apoptosis or autophagy (Gambardella et al., 2020; Read and Schröder, 2021). In ERS, ATF4 acts as a double-edge sword. On the one hand, ATF4 participates in a negative feedback loop and dephosphorylates eIF2α by activating GADD34 and C/EBP homologous protein (CHOP), which leads to the termination of stress response signaling and restoration of protein synthesis. Then, the homeostasis of cells is restored (Figure 1). On the other hand, if severe ER stress persists, ATF4 initiates the activation of caspase 8 expression after the activation of GADD34 and CHOP, which promotes apoptosis (Hetz et al., 2020). However, the reason for GADD34 and CHOP activation by ATF4 in cells in two completely different situations and the specific mechanism are still unclear. In addition, the molecule that plays a selective role in this regulatory process, which is believed to be an important direction of ERS research in the future, remains to be explored.

### 2.2 IRE1α pathway

IRE1α is a type 1 ER transmembrane protein kinase/endonuclease that oligomerizes and autophosphorylates in response to ER stress to activate RNase domains (Credle et al., 2005; Zhou et al., 2006). Under mild UPR conditions, IRE1α promotes the translation of XBP1 mRNA to produce activated XBP1s, a TF that, upon entry into the nucleus, initiates the transcription of a number of genes (Grandjean et al., 2020). In addition, IRE1α activates the ERAD pathway. Moreover, IRE1α RNase can cleave ER-associated mRNA or noncoding functional RNA, which leads to their degradation through regulated IRE1-dependent decay (RIDD). This process results in the regulation of protein folding load, cellular metabolism, inflammation, and inflammasome signaling pathways, both of which alleviate ER stress and promote cell recovery to normal conditions. The IRE1α cytoplasmic domain also serves as a scaffold to recruit adaptor proteins, such as TRAF family members, to activate inflammatory responses under atypical ER stress conditions. (Figure 1). When ER stress is severe and persistent, IRE1α recruits TRAF2. Which further induces the activation of downstream c-Jun N-terminal kinase (JNK) and cell apoptosis. Alternatively, the cascade of caspase 8 or caspase 2 induces apoptosis after the activation of the RIDD pathway (Bashir et al., 2021; Lee et al., 2021).

### 2.3 ATF6 pathway

In response to ER stress, ATF6 is translocated from the ER to the Golgi and cleaved by S1P and S2P to release the basic leucine zipper-containing fragment ATF6p50. ATF6p50 then transports into the nucleus and activates the promoters of UPR target genes, which enhances its capability to process unfolded proteins (Ye et al., 2000). In addition, ATF6 can work with IRE1α to increase the transcription of XBP1 and enhance its capability to degrade unfolded proteins (Figure 1) (Bommiasamy et al., 2009). However, under severe ER stress, ATF6 activates CHOP-mediated apoptosis (Yang et al., 2020b). In recent years, considerable progress has been attained in the field of ERS, and researchers have revealed its key role in the pathophysiological process of diseases. The UPR is an ERS-related signaling pathway that is essential for determining cell fate (cell death or survival) in response to ER stress. Abnormal levels of ERS are closely related to different human diseases, including neurodegenerative diseases, obesity, diabetes, cancer, and autoimmune diseases. However, the mechanism of the survival to death transition under ERS is still unknown. The future challenge is to apply existing research results to develop drugs that can be safely used in clinical practice. To determine the human diseases that can be most effectively treated with these drugs, scholars must study the complexity of ER stress and its interactions with various cellular pathways to shed new light on future diagnostic, preventive, and therapeutic strategies for disease.

## 3 Multidrug-resistant cancers

With the continuous progress of modern medicine, the treatment of malignant tumors has changed greatly. From the earliest surgery or chemotherapy, treatment of malignant tumors has gradually evolved into a comprehensive therapy including surgery, chemotherapy, immunotherapy, molecular targeted therapy, etc. With the proposal and wide application of comprehensive therapy, the survival rate of patients has improved considerably (Siegel et al., 2023). However, tumor MDR greatly affects the prognosis of patients (Nie et al., 2022). Although resistance may develop in response to specific drugs or drug combinations, cross-resistance may confer resistance to drugs with different molecular targets or mechanisms of action. Thus, tumors can develop intrinsic resistance to agents that individuals have never been exposed to (Hanssen et al., 2021). Most deaths of cancer patients are ultimately attributed to tumor MDR (Bukowski et al., 2020). At present, tumor drug resistance is divided into primary and acquired drug resistances. Primary drug resistance refers to the innate resistance of tumor cells to a certain antitumor drug, regardless of whether they have been exposed to the drug. This type of resistance may be caused by the expressions of several mutant genes, abnormal cellular status of tumor cells, or rapid adaptation of tumor cells to the drug. Acquired drug resistance corresponds to the induction of drug resistance in tumor cells during tumor treatment, that is, tumor cells are sensitive to drugs at the initial use and later relapse and develop drug resistance (Chatterjee and Bivona, 2019). Nowadays, tumor MDR is no longer limited to traditional chemotherapy but has shown resistance to immunotherapy and molecular targeted therapy. The mechanism of tumor MDR is extremely complex, and the various mechanisms are not completely independent and often cross. Thus, overcoming this problem should not be limited to a specific or signaling pathway. Combination therapies may be needed to reduce and reverse tumor MDR.

## 4 Mechanism of MDR cancers

### 4.1 Interactions between cancer and drugs

**FIGURE 2:**
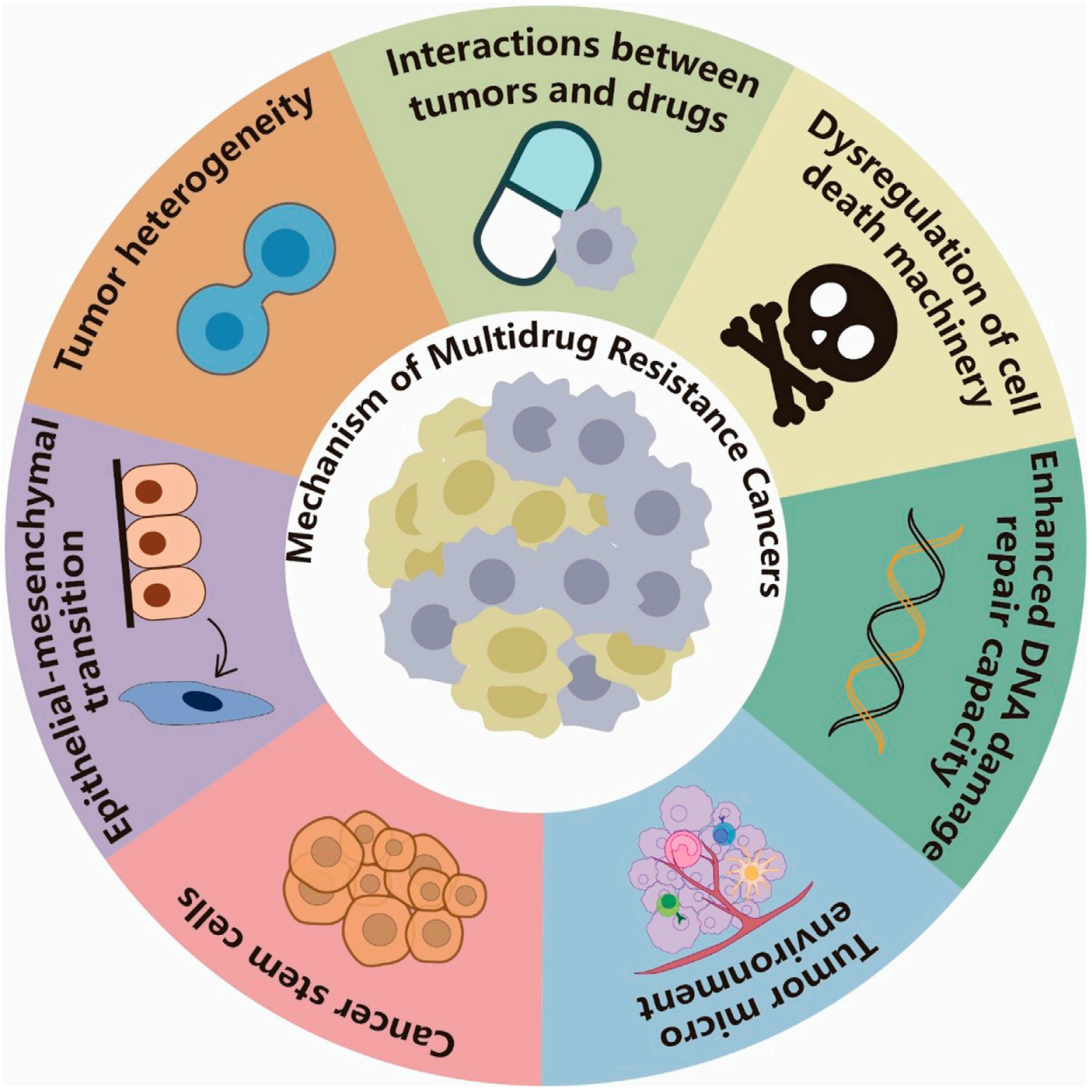
Mechanisms of MDR cancers. Tumor cells can resist drugs by increasing efflux, decreasing absorption, or affecting metabolism. They can also dysregulate the drug-induced cell death mechanism and repair DNA damage caused by chemotherapy drugs, which render the drugs less effective. The TME can mediate tumor MDR through multiple mechanisms, including preventing immune clearance of tumor cells, hindering drug absorption, and stimulating paracrine growth factors to promote cancer cell growth. Cancer stem cells (CSCs) develop MDR mainly through dormancy, epithelial–mesenchymal transition (EMT), MDR, and resistance to DNA damage-induced death. EMT and CSCs often share key signaling pathways and drug resistance phenotypes. EMT induction or EMT TF activation can endow tumor cells with stem cell-like characteristics, and the TME can also promote EMT-induced drug resistance. Tumor heterogeneity is usually a result of chromosomal instability, mutation, and epigenetic changes and considered one of the important reasons for tumor MDR.

Tumor cells can resist chemotherapeutic drugs by increasing efflux, decreasing absorption, or affecting metabolism (Figure 2). The Atp-binding cassette (ABC) transporter family is widely involved in the resistances of various tumor drugs as a drug efflux pump. It uses the energy of ATP hydrolysis to transfer chemotherapy drugs from cells to the outside of cells, reduce the concentration of intracellular drugs, and promote cell resistance. Out of the 48 ABC transporters, 19 can efflux anticancer drugs. ABC subfamily B member 1 (ABCB1), ABC subfamily C member 1 (ABCC1), and ABC subfamily G member 2 (ABCG2) are associated with MDR. They are also known as MDR-related proteins because they can nonspecifically efflux anthracyclines, taxanes, vinca alkaloids, tyrosine kinase inhibitors (TKIs), and other chemotherapy drugs. P-Glycoprotein (ABCB1) was the first identified and is the most studied protein. P-Glycoprotein is generally expressed in normal and tumor epithelial tissues, such as the brain, adrenal cortex, liver, kidney, and intestine. It is mainly responsible for the transport of compounds in a variety of structures but is highly expressed in numerous types of multidrug-resistant cancer cells. ABCC1 is also widely distributed in the kidney, adrenal gland, lung, pancreas, muscle, intestine, thyroid, and prostate. It can transport glutathione disulfides (such as cytotoxic drugs that bind to glutathione) and pump them out of cells. ABCC1 also plays a role in cellular redox homeostasis (Robey et al., 2018; Liu, 2019). On the one hand, it plays a role in protecting normal cells by transporting substrates across the biofilm. However, on the other hand, this property also allows them to become an umbrella for tumor cells.

Downregulation or abnormal binding of uptake transporters and changes in the tumor microenvironment (TME) may lead to decreased drug uptake. Mutations in the folate carrier gene of patients with acute lymphoblastic leukemia can reduce the binding of methotrexate to the transporter and lead to methotrexate resistance in patients (Wojtuszkiewicz et al., 2015). Platinum drug resistance is often caused by reduced drug accumulation of copper transporter 1 (CRT1), a member of the SLC subfamily 31, and several clinical studies have shown that decreased CTR1 expression is associated with reduced concentrations of platinum compounds in tumors and poor prognosis in patients with solid cancers treated with platinum therapy (Kalayda et al., 2012).

The mechanism of action of chemotherapeutic drugs can also rely on the metabolism of enzymes to exert anticancer effects. The upregulation of enzymes involved in drug metabolism can increase the rate of drug decomposition and reduce the efficacy, which leads to MDR. The overexpression of human cytochrome P450 CYP1B1 has been observed in a variety of malignant tumors. However, detecting this protein in normal tissues is difficult. The presence of CYP1B1 in cells reduces the sensitivity of tumors to anticancer drugs. Drug resistance of tumor cells can be reversed by coincubation of cells with the anticancer drug docetaxel (DTX) and cytochrome P450 CYP1 inhibitors (McFadyen et al., 2001).

### 4.2 Dysregulation of the cell death machinery

The cytotoxicity of antineoplastic drugs depends primarily on their capability to induce cell death. The main mechanisms of cell death comprise apoptosis, necrosis, and autophagy-related cell death. Chemotherapeutic agents induce cell death through a variety of molecular and cellular mechanisms, such as reactive oxygen species (ROS) induction, DNA damage, activation of proapoptotic receptors, induction of autophagy-related cell death, and immune cell effector responses. However, cancer cells undergo constant evolution and adaptation, which confer them the ability to evade cell death (Figure 2) (Assaraf et al., 2019). Apoptosis can be activated by a mitochondrial pathway controlled by interactions between the proapoptotic and antiapoptotic members of the BCL2 family; the Bcl-2 antiapoptotic molecule is frequently upregulated in multidrug-resistant tumor cells to prevent drug-induced apoptosis (Maji et al., 2018; Zhang et al., 2021a). The P53 tumor suppressor gene is one of the most studied tumor suppressor genes and the most frequently mutated. It activates proapoptotic proteins, which, when mutated, prevent cell apoptosis or death, reduce sensitivity to antineoplastic drugs, drive cancer metastasis, and enhance MDR during cancer treatment (Yang et al., 2015; Cao et al., 2020). Numerous p53-regulated microRNAs (miRNAs) have been proposed to be involved in p53-regulated tumor functions. MiRNAs play an important role in cancer development, metastasis, angiogenesis, and MDR (Peng and Croce, 2016; Lampis et al., 2020). The miR-17-92 cluster is a novel target for p53-mediated transcriptional repression under hypoxic conditions. When down-regulating this cluster sensitizes cells to hypoxia-induced apoptosis, whereas its overexpression inhibits apoptosis. Therefore, tumor cells with increased miR-17-92 expression may escape hypoxia-induced apoptosis. The above results suggest that p53 and its regulated miRNA form a network, and cancer cells with dysregulated p53 or its target miRNA may have the ability to resist cell death, which results in MDR (Yan et al., 2009). Autophagy refers to the process by which cytoplasmic components are transported to lysosomes for extensive degradation in response to intracellular and extracellular stresses; this process is essential for cell survival in response to hypoxia, genomic instability, ER stress, and nutrient deprivation (Glick et al., 2010; Parzych and Klionsky, 2014). Autophagy often leads to MDR in tumors. On the one hand, autophagy removes damaged proteins and organelles from cancer cells to provide energy for their survival against anticancer therapy and avoid tumor cell death (Cordani and Somoza, 2019). On the other hand, autophagy promotes tumor cells to evade immune surveillance, which also allows tumor cells to survive (Gao et al., 2022). Therefore, autophagy participates in and promotes the development of tumor MDR, helps cancer cells escape apoptosis, and protects tumor cells from chemotherapy and targeted drugs (Onorati et al., 2018; Ferreira et al., 2021). According to some scholars, sustained autophagy activation leads to the turnover of proteins and organelles beyond the survival threshold, which can kill certain cancer cells with a high apoptosis threshold and thereby improve the therapeutic effect. However, to date, *in vivo* evidence of autophagic cell death in mammals is relatively limited; regardless the induction of drug-resistant tumor cell death by autophagic death remains an attractive but remains to be investigated therapeutic strategy (Yang et al., 2011; Choi, 2012).

### 4.3 Enhanced DNA damage repair capacity

Tumor cells may develop drug resistance through the enhancement of DNA damage repair pathways (Figure 2). Anticancer drugs, such as topoisomerase inhibitors, anthracyclines, and cisplatin, can induce different forms of DNA damage. Cisplatin exerts anticancer effects by inducing DNA double-stranded breaks (Kizek et al., 2012; Liu et al., 2019). After their chemotherapy drug-induced DNA damage, tumor cells activate a DNA repair mechanisms, which leads to the development of drug resistance due to the ability of cancer cells to repair DNA damage caused by chemotherapy drugs; this condition reduces the effectiveness of chemotherapy drugs (Trenner and Sartori, 2019). The role of base excision and mismatch repairs in 5-fluorouracil (5-FU) resistance has been demonstrated in numerous studies. At present, researchers are investigating the involvement of other repair pathways in the MDR of tumor cells, and a number of pathways, including homologous recombination (HR) and nonhomologous end joining pathways, have been discovered. Patients who initially respond to cisplatin treatment usually develop drug resistance due to the activation of HR DNA repair mechanisms (Sethy and Kundu, 2021; Sun et al., 2022b). YTH N6-methyladenosine RNA binding protein 1 (YTHDF1) is a m6A-binding protein that promotes the growth of breast cancer cells *in vitro* and *in vivo*. YTHDF1 promotes DNA replication and damage repair; thus, knockdown of YTHDF1 re-sensitizes breast cancer cells to doxorubicin, cisplatin, and olaparib (Sun et al., 2022b). Moreover, C-Jun activation domain-binding protein 1 (Jab1) positively regulates the DNA repair protein Rad51 in a p53-dependent manner. Moreover, the overexpression of Rad51 confers cell resistance to adriamycin and cisplatin in JAB1-deficient cells, and metformin can inhibit cisplatin-mediated upregulation of RAD51 by reducing the stability of RAD51 protein and increasing its ubiquitination, which improves cisplatin resistance (Lee et al., 2019; Liu et al., 2019). Tamoxifen-resistant breast cancer cells exhibit high-level expressions of BARD1 and BRCA1 genes, which are thought to contribute to the MDR of tumor cells to DNA-damaging chemotherapy drugs, including cisplatin and doxorubicin. Silencing BARD1 or BRCA1 expression or inhibition of BRCA1 phosphorylation by dinaciclib restores the sensitivity of tamoxifen-resistant cells to cisplatin (Zhu et al., 2018).

### 4.4 TME

The microenvironment around normal human tissues is an important barrier against tumors and can effectively inhibit tumor growth. Tumor cells colonizing normal tissues can change the microenvironment around tumor cells and form the TME by recruiting cancer-associated fibroblasts (CAFs), which regulate immune cells and their secreted factors and form neovascularization using vascular endothelial cells (Wu and Dai, 2017). Current studies have shown that the TME may mediate tumor MDR through various mechanisms, including preventing immune clearance of tumor cells, hindering drug absorption, and stimulating paracrine growth factors to promote cancer cell growth (Figure 2). The TME is generally composed of three parts: matrix components, cellular components, and soluble factors (Vasan et al., 2019). Tumor-associated fibroblasts are the main stromal components of the TME, and their high density in solid tumors increases tumor interstitial fluid pressure and hinders drug absorption. Several studies has also demonstrated the presence of CAFs in TME-mediated resistance, such as in MDR-associated esophageal squamous cell carcinoma patients, pancreatic cancer patients resistant to gemcitabine, and gastric cancer patients resistant to 5-FU (Assaraf et al., 2019; Fiori et al., 2019; Wu et al., 2021). The tumor immune microenvironment is a subtopic in the study of the TME and has received extensive attention due to its participation in a wide variety of tumor biological processes. Immunosuppressive cells in the TME mainly include Treg cells, tumor-associated macrophages (TAMs), and MDSCs. These cells inhibit the activation, proliferation, and killing function of CD8^+^ T cells by expressing coinhibitory molecules and secreting immunosuppressive factors, which eventually lead to the immune escape of tumor cells (Mao et al., 2021). Treg cells can inhibit the proliferation and activation of effector CD8^+^ T cells through the expression of CD25, competitive binding of interleukin (IL)-2, secretion of IL-10 and transforming growth factor (TGF)-β, and other pathways that promote immune escape and lead to immunotherapy resistance (Sasidharan Nair and Elkord, 2018). TAMs can induce and maintain the immunosuppressive state of the TME through various pathways, such as the expression of immune checkpoint molecules (programmed death-ligand 1), production of immunosuppressive factors (TGF-β and IL-10), secretion of chemokines (CCL17 and CCL22), and abnormal metabolism of amino acids (Mantovani et al., 2017). A large number of soluble cytokines in the TME also enable tumors to evade immune surveillance. Soluble factors, such as TGF-β, vascular endothelial growth factor, chemokines, and inflammatory cytokines, constantly change and interact with each other to induce a complex network of changes. They jointly trigger functional changes in immune and tumor cells and participate in the induction of angiogenesis and interstitial fibrosis in the TME to promote its immunosuppressive nature. Thus, it leads to biological behaviors, such as malignant proliferation, invasion, metastasis, and drug resistance of tumors (Batlle and Massagué, 2019; Khalaf et al., 2021).

### 4.5 CSCs

CSCs are subsets of cancer cells with self-renewal and multidirectional differentiation properties. The CSCs theory states that tumor growth is driven by a small number of CSCs hidden in the cancer. This theory explains why tumor recurrence, metastasis, and MDR are almost inevitable after the initial successful chemotherapy or radiotherapy (Figure 2). This subset has been detected in most blood systems and solid tumors (Batlle and Clevers, 2017; Walcher et al., 2020). CSCs not only exhibit strong proliferation and differentiation abilities but are also considered a source of tumor heterogeneity (Nassar and Blanpain, 2016). Aldehyde dehydrogenase (ALDH) is a selective marker of CSCs in breast cancer, bladder cancer, embryonic rhabdomyosarcoma, head-and-neck squamous cell carcinoma, and lung cancer, and its high expression of ALDH causes resistance to a variety of chemotherapy and targeted agents, such as cisplatin, etoposide, fluorouracil, and gefitinib (Ginestier et al., 2007; Huang et al., 2013). According to current studies, CSCs mainly cause MDR through dormancy, EMT, MDR, and anti-DNA damage-induced death (Phi et al., 2018). After tumor formation, CSCs are considered quiescent or in G0 phase (Schmidt-Kittler et al., 2003). Chemotherapy in the traditional sense often causes irreversible damage to dividing tumor cells by interfering with or inhibiting DNA or RNA synthesis or inhibiting key enzymes required for DNA synthesis. However, most CSCs are in the G0 stage and are thus insensitive to chemotherapy drugs. In such cases, the use of chemotherapeutic drugs usually results in the elimination of tumor cells but enriches CSCs and hence allows the development of MDR (Kurtova et al., 2015). Nestin-ΔTK-IRES-GFP transgenic mice were used to label resting CSCs and glioma tumor cells in a mouse glioma model. After temozolomide treatment, the dividing tumor cells were effectively killed, but GFP-labeled resting CSCs proliferated rapidly (Chen et al., 2012). In addition, the ABC transporter family is involved in the MDR of CSCs, and a considerable number of ABC transporters are commonly overexpressed in cancer, especially in CSCs. CSCs alter DNA damage response (DDR) and repair pathways, and an efficient DDR leads to frequent radiation and chemical multiresistance (Huang et al., 2020).

### 4.6 EMT

EMT plays an important role in cancer progression, metastasis, and drug resistance (Figure 2). In this process, epithelial cells lose their apical‒basal polarity and cell‒cell adhesion and transfer to invasive mesenchymal cells, and although the specific links surrounding EMT and cancer metastasis remain to be further studied, the role of EMT in cancer drug resistance has been increasingly recognized (Du and Shim, 2016; Shibue and Weinberg, 2017). In the early 1990s, Sommers et al. observed EMT in two adriamycin-resistant MCF-7 cell lines and one vinblastine-resistant ZR-75-B-cell line (Sommers et al., 1992). Subsequently, studies have revealed that multidrug-resistant tumors, including pancreatic cancer, bladder cancer, breast cancer, etc., are often accompanied by EMT, and the signaling pathways that promote the EMT phenotype lead to MDR of tumors (Du and Shim, 2016). The EMT-mediated aggressive behavior of cancer cells can lead to tumor resistance to paclitaxel (PTX) and DTX. In this case, the upstream mediators of EMT, such as zinc finger E-box binding homeobox (ZEB)1/2, TGF-β, and miRNAs, are involved in regulating the response of cancer cells to PTX and DTX. On the contrary, the sensitivity of cancer cells to PTX and DTX can be restored after the inhibition of EMT by tumor suppressors (Ashrafizadeh et al., 2021). In lung cancer cells, gefitinib treatment can activate NOTCH-1 signaling, which results in an acquired EMT phenotype and treatment resistance (Xie et al., 2012). Although the link between EMT and multidrug-resistant tumors has been reported for a long time, the involved mechanisms remain elusive. One such mechanism is the remarkable similarity between the signaling pathways activated during EMT and those of CSCs (Huber et al., 2005). ZEB1 is a TF associated with EMT and can regulate CSCs self-renewal and drug resistance by regulating O-6-methylguanine methyltransferase via miR-200c and c-MYB (Siebzehnrubl et al., 2013). CSCs express various markers of normal stem cells, including their ability to survive in a dedifferentiated state (Gupta et al., 2019). However, cells undergoing EMT also have stem-like characteristics, and EMT and stem cell markers are co-expressed in tumor cells of patients with tumor metastasis (Oskarsson et al., 2014). In addition, EMT induction or activation of EMT TFs endows tumor cells with stem-like characteristics (Puisieux et al., 2014). Altogether, EMT and CSCs often share key signaling pathways and drug-resistant phenotypes. Cells undergoing EMT similarly overexpress ABC transporters and consequently develop MDR (Du and Shim, 2016). The TME is also a factor mediating EMT-driven drug resistance. Hypoxia is another important TME that promotes cancer cells to undergo EMT and acquire drug resistance. The activation of hypoxia-inducible factor (HIF)-1α under hypoxic conditions promotes EMT in hepatocellular carcinoma (HCC) and induces drug resistance by increasing the expression of MDR1. Knockdown of HIF-1α reversed the EMT phenotype and abolished the drug resistance phenotype of HCC under hypoxic conditions, which further confirmed the role of hypoxia/HIF-1α in EMT-driven drug resistance (Jiao and Nan, 2012).

### 4.7 Tumor heterogeneity

Tumor heterogeneity refers to the changes in molecular biology or genes during tumor evolution, which lead to differences in the growth rate, invasion ability, and drug sensitivity of different tumor cells in the same tumor (Figure 2) (McGranahan and Swanton, 2017). Tumor heterogeneity can be manifested as spatial and temporal heterogeneities. Spatial heterogeneity describes the uneven distribution of genetically diverse tumor subsets in different disease sites or within a single disease site or tumor (Graf and Zavodszky, 2017). Temporal heterogeneity indicates the occurrence of tumors in patients at different stages, which may face different biological selection pressures; as a result, dynamic changes in individual genetic diversity occur over time (Dagogo-Jack and Shaw, 2018). Most tumors are complex ecosystems that evolve in under strong selection pressures from the microenvironment, including nutritional, metabolic, immune, and therapeutic components. A strong selection pressure promotes the diversification of malignant and benign (i.e., endothelial, mesenchymal, and immune) components of the TME, which eventually leads to a certain degree of tumor heterogeneity. This leads to aggressive disease progression and treatment resistance (Vitale et al., 2019). Chromosomal instability, inherited missense mutations, or epigenetic changes such as DNA methylation or histone modifications, can contribute to changes in tumor heterogeneity (Gorre et al., 2001; Stratton et al., 2009; Negrini et al., 2010). Tumor heterogeneity promotes MDR to epidermal growth factor receptor (EGFR) TKIs in lung cancer. In targeted therapy, the resistance gene mutations caused by tumor heterogeneity exhibit complexity. The difference in resistance mutation sites in the anaplastic lymphoma kinase (ALK) gene between different patients is complicated. Examples include G1202R, G1269A, L1196M, and F1174C (Katayama, 2017; Cooper et al., 2022), which can also exist as comutations of multiple drug resistance sites in the same patient, such as ALK and EGFR-L858R or ALK and BRAF comutation (Kim et al., 2013; Urbanska et al., 2020). Therefore, can targeting multiple signaling pathways in combination with antitumor strategies reduce the development of multidrug-resistant tumors?

## 5 Links between ERS and multidrug resistant tumors

Whether ERS is a favorable or an unfavorable factor for tumor MDR needs more research. At present, a huge number of evidence indicates the persistence of ERS in a variety of cancer types. However, whether ERS promotes or inhibits the development of patient tumors has been vigorously debated. On the one hand, the UPR is an adaptive response during ERS, and it relists the ER load by reducing the protein source and increasing the route of protein to restore cell homeostasis. Tumor cells can use this feature to promote the progression of malignant tumors and MDR. On the other hand, when overactivated ERS exceeds the threshold that cancer cells can withstand, it will activate proapoptotic pathways and induce cancer cell death (Oakes, 2020). Similar to the abovementioned mechanisms of tumor drug resistance, the mechanisms of ERS leading to tumor MDR are extremely rich and are often considered to be related to enhanced tumor drug excretion, which prevents the apoptosis of tumor cells through chemotherapy drugs, resistance to death through miRNA, and protective cell autophagy.

### 5.1 GRP78 and multidrug-resistant tumors

Three UPR-related transmembrane proteins are inactivated by GRP78 before ERS. During stress, the amount of unfolded protein in the ER lumen increases, which triggers the dissociation of the three protein receptors from GRP78. Thus, GRP78 can be considered as the initiating part of the entire ERS. GRP78 is a major ER molecular chaperone with Ca2+-binding and anti-apoptotic properties (Luo et al., 2006) and is therefore often considered to be associated with MDR in tumors. Compared with that in normal tissues, the expression of GRP78 is increased considerably in liver cancer, gastric cancer, breast cancer, renal cell carcinoma, and other tumors (Luo and Lee, 2013). GRP78 induces tumor MDR mainly through the following two pathways. On the one hand, it can reduce ER stress and cell apoptosis and therefore increase the resistance of tumor cells to chemotherapy and radiotherapy. GRP78 blocks cell apoptosis by binding and inactivating apoptotic components; GRP78 can bind BIK and caspase-7 in the ER and inhibit the cell apoptosis induced by CHOP (Reddy et al., 2003; Fu et al., 2007). On the other hand, GRP78 on the cell surface (sGRP78) transmits signals to promote the EMT and stemness of cancer cells, which results in MDR (Conner et al., 2020; Xia et al., 2021). In pancreatic cancer, downregulation of GRP78 reduced the clonogenic and self-renewal properties of pancreatic cancer cell lines *in vitro* (Dauer et al., 2019). Similarly, an increased level of GRP78 was found in gefitinib-resistant lung cancer cells, accompanied by the increase in EMT and CSCs characteristics (Liao et al., 2020). ER ribosome-binding protein 1 can enhance the expression of GRP78 and makes lung cancer cells resistant to various chemotherapy drugs, such as tunicamycin and doxorubicin (Tsai et al., 2013). The overexpression of GRP78 can also attenuate the activation of caspase-4 and caspase-7 and the induction of apoptosis by drugs, which leads to the resistance of melanoma to cisplatin and doxorubicin (Jiang et al., 2009). In addition, GRP78 has been identified as a positive regulator of the acquisition of sorafenib resistance in hepatocytes and a major target for overcoming sorafenib resistance (Chiou et al., 2010). Numerous chemotherapy drugs combined with UPR inhibitors or activators can prevent cytoprotection and induce apoptosis, restore the sensitivity of cancer cells to chemotherapy drugs, and improve the efficacy of chemotherapy drugs. Given its importance in cancer cell resistance, GRP78 has also been a major target of anticancer therapy. The inhibition of glutamine-fructose-6-phosphate aminotransferase activity downregulates GRP78 expression and activates IRE1α, which leads to the increased sensitivity of non-small-cell lung cancer (NSCLC) cells to cisplatin and further initiates the apoptotic pathway (Chen et al., 2019). In pancreatic cancer cells, the combined treatment with siGRP78 (small-interfering RNA (siRNA) for GRP78) reduced the percentages of chemotherapeutic drug efflux to 27.1% and 2.9%–0.56% and 0.68%, respectively, compared with that of gemcitabine or PTX alone. This process is mediated by ABC transporters and regulated by the TF NRF2. NRF2 is a downstream target gene of PERK, and its activation upregulates the expressions of stress response proteins, drug metabolism enzymes, and ABCB1-encoded MDR1, which reverse the MDR of tumors (Salaroglio et al., 2017). Similarly, the inhibition of GRP78 expression by siRNA increases the apoptosis and sensitivity of breast cancer cells to chemotherapy and restores the anti-estrogen sensitivity of drug-resistant breast cancer cells (Yang et al., 2020a). In laryngeal squamous cell carcinoma (LSCC), the overexpression of miR-936 can substantially reduce the protein level of GPR78, inhibit the proliferation, migration, and invasion of LSCC cells, and improve sensitivity to doxorubicin and cisplatin (Lin et al., 2020). Overall, GRP78 has been extensively studied in multidrug-resistant tumors, and the findings identify GRP78 as a novel therapeutic target against MDR to chemotherapy in cancer cells.

### 5.2 PERK pathway and multidrug-resistant tumors

PERK often plays a key role in inducing apoptosis, which implies its close relationship with cancer treatment and drug resistance. PERK activates CHOP by modulating ATF4 expression through trans-autophosphorylation and phosphorylation of eIF2α after the dissociation of GRP78. CHOP promotes protective autophagy, which leads to drug resistance by inhibiting mammalian target of rapamycin (mTOR) complex 1 and promoting the expression of the ATG5–ATG12–ATG16L complex. CHOP is also a mediator of apoptosis. miR-146a induces drug resistance by inhibiting CHOP-mediated apoptosis. P-Glycoprotein, whose level increases after PERK activation, pumps several intracellular drugs out of tumor cells, which in turn reduces drug-induced apoptosis of tumor cells and leads to drug resistance (Cao et al., 2021). In terms of inhibiting tumor apoptosis, insulin resistance can lead to 5-FU resistance in HCC through activation of the PERK pathway and upregulation of Bcl-2 anti-apoptotic protein (Liu et al., 2016). In addition, the treatment of renal cell carcinoma cell lines with sunitinib *in vitro* increases GRP78 expression, which promotes the proliferation of renal carcinoma cells under hypoxia/hypoglycemia stress and resistance to apoptosis by stimulating PERK/eIF2α signaling (Correia de Sousa et al., 2023). In enhancing resistance to antitumor drug efflux, gene profiling demonstrated high levels of PERK in chemotherapy-resistant human colon cancer cells by. The related study further revealed a link between PERK and the nuclear receptor and TF Nrf2, which directly regulates the transcription of the ABC cassette transporter multidrug resistance protein 1 (MRP1). Targeting the PERK/Nrf2/MRP1 axis eliminated resistance to chemotherapy (Salaroglio et al., 2017). In terms of interaction with miRNA, the ATF4/PERK pathway also interacts with the long noncoding RNA (lncRNA) ZFAS1 signaling pathway, which is important in sorafenib resistance. Sorafenib, which is thought to resensitize cells to itself, may promote ZFAS1 activation by activating the PERK/ATF4 pathway and inhibiting PERK signaling in resistant HCC cells (Lin et al., 2021a). In addition, Golgin A2 pseudogene 10 (GOLGA2P10) is a pseudogene-derived lncRNA and is a nonfunctional residue formed during the evolution of a gene family. Pseudogenes are similar to normal genes but without normal function; they often exist in multiple gene families in eukaryotes. GOLGA2P10 is frequently upregulated in HCC tissues, induced by PERK/ATF4/CHOP signaling, and protects tumor cells from ER stress-induced apoptosis by regulating members of the Bcl-2 family of antiapoptotic proteins (Wu et al., 2020). In terms of drug resistance caused by CSCs, histone methyltransferase G9a is a potential target for epigenetic therapy of acute myeloid leukemia, and PERK/NRF2 signaling plays a key role in protecting leukemia stem cells (LSCs) from ROS-induced apoptosis, which confers LSCs with resistance to G9a inhibitors. Treatment with PERK/NRF2 or autophagy inhibitors overcomes the resistance to G9a inhibition and eliminates LSCs (Jang et al., 2020). Oral squamous cell carcinoma (OSCC) cells with high expression of CD10 also have CSC-related characteristics, which in turn affect tumor growth, EMT, and cisplatin resistance. CD10-positive cells secrete IL8 and promote cisplatin resistance in OSCC through the PERK signaling pathway (Pu et al., 2021). PERK promotes the binding of ATF4 to TRB3 through eIF2α phosphorylation, which inhibits the AKT/mTOR axis and increases basal autophagy in melanoma cells. The activation of the PERK-CHOP axis also promotes the transcription of autophagy genes through cooperation with ATF4 and C/EBPβ (Kong et al., 2022). The inhibition of PERK-dependent ERS using the PERK inhibitor GSK2606414 abolishes the resistance caused by BRAF-induced autophagy in melanoma cells (Ma et al., 2014). Similarly, vemurafenib induces resistance through autophagy in BRAF-mutant thyroid cancer cell lines. This autophagy is associated with the induction of eIF2α phosphorylation and CHOP expression by vemurafenib; in addition, autophagy inhibitors can effectively enhance the antitumor activity of vemurafenib in thyroid cancer and are tolerated well *in vivo* (Wang et al., 2017). One of the reasons for the high mortality rate of osteosarcoma is its easy resistance to chemotherapy drugs. Sestrin2, one of the most important cellular stress proteins, is highly expressed in surviving osteosarcoma cells after chemotherapy. Sestrin2 activates autophagy by inhibiting mTOR through the PERK–eIF2α–CHOP pathway and inhibits apoptosis through Bcl-2. In addition, a low sestrin2 expression can effectively reduce autophagy, increase p-mTOR expression, and reduce Bcl-2 expression in human osteosarcoma cells after chemotherapy of NU/NU mice. It can also promote the apoptosis of osteosarcoma cells (Tang et al., 2021). In a study related to pancreatic cancer, the use of TGF-β1 or cobalt chloride to simulate a severe hypoxic environment induced EMT in pancreatic ductal adenocarcinoma cells, and further treatment with acriflavine inhibited this conversion process. Gene enrichment analysis showed that by blocking eIF2a phosphorylation and reducing ATF4 translation, acriflavine inhibited the unfolded protein-responsive PERK/eIF2a/ATF4 pathway, that is, acriflavine restored the drug sensitivity of acquired drug-resistant pancreatic cancer cell lines. Therefore, targeting the PERK/eIF2a/ATF4 pathway can be used to inhibit EMT in pancreatic cancer cells. In return, the drug resistance of tumors can be reversed (Dekervel et al., 2017).

### 5.3 IRE1α pathway and multidrug -resistant tumors

As the most conserved signaling pathway in the UPR, IRE1α participates in the regulation of various stages of tumor development, and XBP1 plays an irreplaceable role in promoting cell survival and tumor MDR. Therefore, the close regulation of the IRE1α pathway can be used as an effective treatment of multidrug-resistant tumors (Shi et al., 2019). IRE1α is activated upon dissociation from GRP78, and the downstream XBP1 upregulates heat shock factor 1 (HSF1) expression. Meanwhile, HSF1 upregulates the expression of Bcl2-associated athanogene-3and stabilizes the expression of the antiapoptotic protein Mcl-1, which inhibits cell apoptosis and leads to drug resistance. In addition, IRE1α and ATF6 promote the expression of XBP1 and downstream HSF1. HSF1 promotes Bcl-1 expression through the receptor-interacting serine/threonine-protein kinase 1-mitogen-activated protein kinase (MAPK) 8/9 axis to induce protective autophagy, which leads to drug resistance. IRE1α also triggers Wnt signaling and nuclear factor (NF)-κB to promote tumor cell survival, which in turn reduces drug-induced apoptosis and results in MDR (Cao et al., 2021). Anticancer drugs, such as 5-FU, can activate the IRE1α-XBP1 pathway to induce the expressions of ABCB1, ABCC1 and ABCG2 in colon cancer cells. The inhibition of IRE1α RNase activity with the small-molecule 4μ8c suppresses the drug-induced expression of these ABC transporters and resensitizes 5-FU-resistant colon cancer cells to drug treatment (Gao et al., 2020). The overexpression of activated protein kinase (RACK1) prevents the apoptotic effect of sorafenib on HCC cells by upregulating XBP1 (Zhou et al., 2015). In addition, IRE1α can cleave and regulate miRNA (Kim and Croce, 2021), which is particularly interesting in the context of drug resistance. miRNA upregulation can confer resistance to 5-FU in HCC cell lines. miR-122 specifically targets the membrane transporter SLC7A1 that is associated with sorafenib resistance. The upregulation of miR-122 may reduce SLC7A1 expression and resensitize HCC cells to sorafenib treatment (Khaled et al., 2022). Sorafenib exposure of HCC cells can upregulate the IRE1α signaling pathway to induce autophagy. *In vitro* and *in vivo* studies showed that HCC cells were resensitized to ER stress-induced cell death when autophagy was inhibited (Shi et al., 2011). Tamoxifen has been widely used to reduce estrogen receptor (EsR)-positive breast cancer patients; however, approximately half of EsR-positive breast cancer patients exhibit chemotherapy resistance. XBP1s expression is highly correlated with EsR-positive breast cancer patients, and STF-083010 is an XBP1 splicing inhibitor. It can reverse the sensitivity of drug-resistant cells to tamoxifen (Shi et al., 2019). EsRβ reduces tumor survival in antiestrogen-sensitive and antiestrogen breast cancer cells. Some scholars believe that the upregulation of EsRβ can inhibit the expressions of IRE1 and XBP1, increase the sensitivity of tumor cells to tamoxifen, and cause the apoptosis of chemoresistant cells (Rajapaksa et al., 2015). In normal cells, IRE1 activity is impaired under sustained ERS, which leads to PERK-mediated apoptosis (Lin et al., 2007); however, in melanoma cells, IRE1 activity is maintained through the MEK/ERK pathway, which counteracts the PERK-mediated apoptosis (Tay et al., 2014). Meanwhile, PERK/eIF2α/ATF4 signaling protects chemotherapy-resistant hypoxic cells by inducing glutathione synthesis and reducing ROS accumulation. Activated ERS may also activate NF-κB and inhibitor of apoptosis (IAP) through the IRE1α–TRAF2 pathway, which leads to MDR; therefore, the use of IAP antagonists can enhance the effectiveness of melanoma therapy (El-Khattouti et al., 2016; Bai et al., 2021; Kong et al., 2022). The efficacy and safety of apatinib in the treatment of advanced gastric cancer and other tumors have been confirmed. Apatinib can induce autophagy in colorectal cancer cell lines through ERS, specifically through the IRE1α signaling pathway. Apatinib-induced protective autophagy has been considered a possible new drug resistance mechanism, and blocking autophagy can promote apoptosis in apatinib-treated colorectal cancer cell lines (Cheng et al., 2018). Sunitinib triggers protumor NF-kB activity through the IRE1α/TRAF2/IKKβ signaling axis, which promotes cell survival (Makhov et al., 2018). Tumor cells experiencing ERS can continue to propagate such condition in tumor-infiltrating leukocytes through paracrine signalling (Jiang et al., 2020), which contributes to the malignant progression and immune tolerance of the host and promotes drug resistance to immunotherapy. Cytokines in the TME, such as IL-4, IL-6, and IL-10, can lead to drug resistance through signal transducer and activator of transcription (STAT)3/6 activation of the IRE1α–XBP1 branch of macrophages (Yan et al., 2016). In addition, increased ROS production in tumor-infiltrating dendritic cells (TDCs) excessively activates IRE1/XBP1. Furthermore, such condition affects lipid metabolism and leads to the abnormal accumulation of liposomes and decreased ability of TDCs to cross-present antigen to T cells. This is also one of the reasons for tumor escape. Silencing XBP1 in TDCs using siRNA can restore its immunostimulatory activity *in situ* in immunotolerant TDCs (Cubillos-Ruiz et al., 2015). In conclusion, the IRE1α signaling pathway plays an irreplaceable role in MDR to chemotherapy and immunotherapy.

### 5.4 ATF6 pathway and multidrug -resistant tumors

ATF6 is the most mysterious of the three pathways, and the relationship between ATF6 and tumor MDR has not been widely explored. ATF6 can activate GRP78, which inhibits caspase-3 activation, maintains the stability of the ER and the internal environment, and leads to tumor resistance (Shen et al., 2002; Dong et al., 2004). The chemoresistance of ovarian cancer is related to the inhibitor of DNA binding 1 (ID1)-induced autophagy. ID1 first activates NF-κB signaling by promoting the nuclear translocation of NF-κBp65, which enhances the expression and secretion of IL-6 in cancer cells. It subsequently activates STAT3 through protein phosphorylation of Y705, which promotes the transcription of ATF6 and subsequently induces ERS stress to promote autophagy. As a result, cancer cells develop resistance to cisplatin and PTX treatment. In addition, patients with high ID1 or ATF6 expression have poor overall and progression-free survival due to resistance to platinum therapy (Meng et al., 2020). Similarly, in tumors of the female reproductive system, ATF6 is highly expressed in cervical cancer cells. The upregulation of ATF6 in cervical cancer cells promotes their proliferation and inhibit apoptosis. ATF6 inhibits autophagy but promotes EMT through the MAPK signaling pathway, which is a possible reason for the chemoresistance of cervical cancer cells. The inhibition of ATF6 can promote apoptosis by inhibiting Bcl-2 and increasing the levels of caspase-3 (Liu et al., 2020). In recent studies related to chemotherapy resistance of gastric cancer, Janus kinase 2/STAT3 inhibitors reduced 5-FU resistance and autophagy through ATF6-mediated ERS (Ma and Wang, 2022). Compared with the other two pathways, studies on the ATF6 signaling pathway are limited, which means that this signaling pathway has great potential for research. Moreover, the ATF6 signaling pathway has shown great value in the study of reversing tumor drug resistance.

### 5.5 Enhancing ERS-mediated proapoptotic pathways in the treatment of multidrug-resistant tumors

The ERS-mediated proapoptotic pathway also offers a promising research direction for the treatment of multidrug-resistant tumors. Under prolonged ERS, the prosurvival function of the UPR is transformed into a proapoptotic signal and executed by mitochondria (Bhat et al., 2017). On the one hand, ER directly activates the apoptotic pathway through ERS-mediated calcium leakage into the cytoplasm, which leads to the activation of death effectors. ATF4, on the other hand, initiates apoptosis upon activation of GADD34 and CHOP. Therefore, the PERK/eIF2α/ATF4 pathway is often considered to play a key role in tumor progression and the development of cancer therapies (Chen et al., 2013; Cheng and Dong, 2018; Li et al., 2019). MCC1734, a derivative of coumarin, showed varying degrees of cytotoxicity against five multidrug-resistant cell lines expressing different resistance mechanisms and could not be pumped out of resistant cancer cells; thus, this compound shows promise in killing multidrug-resistant tumors. MCC1734 possibly exerts antitumor effects by upregulating the p-PERK, eIF2α, ATF4, and CHOP proapoptotic pathways in tumor cells (Lu et al., 2021). Similarly, lobaplatin promotes apoptosis and inhibits the proliferation of HCC by upregulating the PERK-eIF2α-ATF4-CHOP pathway (Li et al., 2019). The abnormal production of secreted mucins (MUCs) is an important feature of pancreatic ductal adenocarcinoma. The overexpressed mucins form a physical barrier to prevent drugs from reaching the target site. The transmembrane mucin MUC-4 is widely involved in the drug resistance in tumor cells. Silencing of MUC-4 gene expression in pancreatic cancer cells increases the rate of cell apoptosis induced by bortezomib through the mitochondrial pathway, which is mediated by the activated CHOP apoptotic pathway (Wissniowski et al., 2012). These results are expected to weaken the resistance of pancreatic cancer, colorectal cancer, and other tumors with a high mucin expression to chemotherapy drugs. In clear-cell renal cell carcinoma (ccRCC), sunitinib-resistant ccRCC cells showed considerably lower death-associated protein kinase 1 (DAPK1) mRNA and protein levels than sunitinib-sensitive ccRCC cells. The overexpression of DAPK1 enhances the apoptosis of sunitinib-resistant ccRCC cells through the ATF6-dependent ERS pathway (Song et al., 2020). Icariside II (IS) exhibits antitumor activity in various cancers, such as liver cancer, breast cancer, prostate cancer, and NSCLC (Xu et al., 2021). The combination therapy involving IS and cisplatin inhibits the proliferation of NSCLC cells and induces apoptosis through the activation of ERS by IS; these actions include the three branches of UPR signaling, namely, PERK, IRE1, and ATF6, and the downstream PERK-eIF2α-ATF4-CHOP pathway, which enhances cisplatin-induced apoptosis (Tang et al., 2022). In diffused large B-cell lymphoma, the overexpression of XBP1 greatly enhances ibrutinib-induced apoptosis in sensitive and resistant cells (Zhang et al., 2021b). Compared with ERS-related prosurvival pathways, studies on proapoptotic pathways are limited, but this does not affect their status and role in tumor MDR. Proapoptotic pathways remain a powerful tool in the addressing the problem of multidrug-resistant tumors.

### 5.6 Nanotherapeutic and multidrug-resistant tumors

In recent years, with the emergence of nanotechnology, nanocarrier drugs for the treatment of multidrug resistant tumors have been developed, and scientists are conducting extensive exploration and research along this direction. Nanomaterials refer to materials in the nanometer range of 1-100 nm, which have unique optical, magnetic and electrical properties (Cheng et al., 2021; Ashrafizadeh et al., 2023). Compared with traditional drugs, nanocarrier drugs can greatly improve the ability to selectively kill tumors and increase the therapeutic effect of drug-resistant tumors. For example, Quercetin still has anticancer effects on adriamycin and docetaxel resistant prostate cancer cells and can reverse drug resistance. Quercetin can reduce the expression of Bcl-2 protein and induce apoptosis of prostate cancer cells by activating IRE1α pro-apoptotic pathway. However, the main problem with quercetin is its low bioavailability and rapid metabolism. Encapsulation of quercetin into the Nano-vehicle agents either *in vivo* or *in vitro* could delay or prevent its metabolism, thereby maintaining high levels of quercetin in blood and other tissues for a long time (Liu et al., 2014; Hussain et al., 2021). At the same time, questions have been raised about the safety of nanomaterials, whether they can reach the target site smoothly and whether they have an effect on normal tissues. Luteinizing-hormone-releasing hormone (LHRH)-conjugated, polyethylene glycolylated (PEGylated), poly-lactide-co-glycolide nanocapsules conjugated to docetaxel and quercetin were formulated by researchers, The capsule was considered to be biodegradable, non-toxic, and able to target prostate cancer, and the results showed that the capsule exhibited reliable anti-tumor activity both *in vitro* and *in vivo* (Shitole et al., 2020). This combination gives us an important hint that chemotherapeutic drugs combined with ERS modulators and paired with nanocarriers may be a new weapon against drug-resistant tumors. In addition, in order to further improve the efficacy of anticancer drugs, the coupling of monoclonal antibodies with cytotoxic drugs, called antibody-drug conjugates (ADCs), has been studied using trastuzumab (Tmab) in ADCs system. The results showed an improved therapeutic effect compared with Tmab alone (Nieto et al., 2020). Ginsenoside is a group of naturally occurring chemicals in ginseng extract. ginsenoside Rg3(Rg3) is one of the well-studied ginsenoside. Rg3 can promote the apoptosis of tumor cells through IRE1α, PERK and ATF6 pathways. Investigators developed a folate-targeted PEGylated cyclodextrin-based nanoparticle to co-deliver Rg3 and quercetin, more surprisingly, combine the resulting compound with anti-PD-L1 antibody achieved chemo-immunotherapy for colorectal cancer (Sun et al., 2022a). However, only a relatively small number of nanodrugs have been well developed and put into clinical use, and researchers still face problems such as low drug loading and premature drug leakage (Fu et al., 2022a). With the development of Artificial intelligence (AI), can nanodrugs be combined with AI technology. AI will be used to target and regulate the movement of nanocarriers and the release of chemotherapy, targeted drugs, immune checkpoint inhibitors and ERS modulators, so as to achieve the purpose of combined anti-multidrug resistant tumor treatment.

## 6 Discussion

At present, resistance to traditional chemotherapy, targeted therapy, or immunotherapy drugs is the most critical factor for tumor resistance or recurrence. Given the universality and complexity of drug resistance mechanisms, when tumor cells become resistant to one drug, they also usually develop different degrees of resistance to other drugs of the same type. Some tumor cells exhibit MDR, which greatly reduces the sensitivity of tumors to drugs and affects patient prognosis. Malignant cells utilize various strategies to proliferate under adverse conditions while suppressing the development of antitumor immune responses; in addition, the continuous activation of ERS sensors confers great tumorigenic, metastatic, and MDR capabilities to malignant cells (Cubillos-Ruiz et al., 2017). Multidrug-resistant tumors are still the main cause of death in cancer patients. Regardless, with the deepening of ERS research, the regulation of ERS prosurvival and proapoptotic pathways has become a tool against multidrug resistant tumors. However, the study of ERS-related multidrug resistant tumors still faces serious challenges, such as how GRP78 senses and measures protein metabolic stress and whether a specific choice should be selected from the three pathways when GRP78 in tumor cells faces the complex TME and generates ERS. It also indicates whether researchers can correctly and precisely select the signaling pathway of ERS leading to drug resistance. Second, researchers can determine whether a threshold exists for cells to identify prosurvival or proapoptotic pathways and whether researchers can detect and modulate that threshold. Thus, when ERS occurs, researchers can downregulate the threshold for tumor cells to switch to the proapoptotic pathway to allow more resistant cells to self-select the proapoptotic pathway and thus reverse tumor resistance. More importantly, when developing related drugs, a drug mechanism that ensures can kill multidrug-resistant cancer cells without affecting normal cells must be determined. Although numerous methods can be used to detect ERS levels *in vitro*, better methods for evaluating ERS *in vivo* are still lacking. Without sensitive and accurate detection indicators, the efficacy of drugs cannot be accurately detected, which results in treatment-related risks and harm. Moreover, compounds targeting ERS modulators can maintain a high degree of specificity and minimize side effects in preclinical or clinical applications. Finally, the types or characteristics of tumors that are prone to ERS-associated MDR remain to be elucidated. Although the research on ERS has achieved extremely rich results, only by dealing with the current bottlenecks can researchers ensure that ERS, which is a tool against MDR, will not become a double-edged sword. Therefore, researchers still need to elucidate the mechanism of ERS in tumor drug resistance and treatment to provide novel ideas for tumor treatment. Finally, the mechanisms of ERS that lead to drug resistance or treatment of drug-resistant tumors are not limited to a certain pathway or mechanism. Mechanisms are often interconnected and interact with each other and form an extremely complex network, which can also become an important direction of ERS research.

## 7 Conclusion

ERS plays an important role in various aspects of tumor MDR, and its mechanism and application are an important but difficult topic in current tumor research. Numerous studies have confirmed that ERS can promote tumor cell death, improve drug efficacy, and reverse drug resistance through synergistic effects with antitumor drugs. The core issue is the targeted regulation of ERS. The in-depth study of ERS also provides possible therapeutic targets for the treatment of multidrug-resistant tumors and new treatment strategies for drug-resistant patients. Before clinical application, a number of problems remain to be solved. Regardless, the in-depth study of ERS and the mechanism of tumor MDR will certainly create new opportunities for the diagnosis and treatment of tumors.
